# Plant-Produced Mouse-Specific Zona Pellucida 3 Peptide Induces Immune Responses in Mice

**DOI:** 10.3390/vaccines11010153

**Published:** 2023-01-10

**Authors:** Khadijeh Ghasemian, Inge Broer, Jennifer Schön, Nadine Kolp, Richard Killisch, Jana Huckauf

**Affiliations:** 1 Department of Agrobiotechnology and Risk Assessment for Bio and Gene Technology, Faculty of Agricultural and Environmental Sciences, University of Rostock, 18059 Rostock, Germany; 2 Department of Reproduction Biology, Leibniz Institute for Zoo and Wildlife Research (IZW), 10315 Berlin, Germany; 3 BIOSERV, Analytik und Medizinprodukte GmbH, 18059 Rostock, Germany

**Keywords:** wildlife population control, immunocontraception, zona pellucida 3, recombinant protein, plant-produced vaccine, *Nicotiana benthamiana*

## Abstract

Contraceptive vaccines are designed to stimulate autoimmune responses to molecules involved in the reproductive process. A mouse-specific peptide from zona pellucida 3 (mZP3) has been proposed as a target epitope. Here, we employed a plant expression system for the production of glycosylated mZP3 and evaluated the immunogenicity of plant-produced mZP3-based antigens in a female BALB/c mouse model. In the mZP3-1 antigen, mZP3 fused with a T-cell epitope of tetanus toxoid, a histidine tag, and a SEKDEL sequence. A fusion antigen (GFP-mZP3-1) and a polypeptide antigen containing three repeats of mZP3 (mZP3-3) were also examined. Glycosylation of mZP3 should be achieved by targeting proteins to the endoplasmic reticulum. *Agrobacterium*-mediated transient expression of antigens resulted in successful production of mZP3 in *Nicotiana benthamiana*. Compared with mZP3-1, GFP-mZP3-1 and mZP3-3 increased the production of the mZP3 peptide by more than 20 and 25 times, respectively. The glycosylation of the proteins was indicated by their size and their binding to a carbohydrate-binding protein. Both plant-produced GFP-mZP3-1 and mZP3-3 antigens were immunogenic in mice; however, mZP3-3 generated significantly higher levels of serum antibodies against mZP3. Induced antibodies recognized native zona pellucida of wild mouse, and specific binding of antibodies to the oocytes was observed in immunohistochemical studies. Therefore, these preliminary results indicated that the plants can be an efficient system for the production of immunogenic mZP3 peptide, which may affect the fertility of wild mice.

## 1. Introduction

Rodents are among the most important vertebrate pests in many parts of the world, causing economic, social, and environmental costs and damage [1,2]. Mice, in particular, are considered serious agronomic pests both in the preharvest and postharvest stages [3,4]. Moreover, they can endanger public health by spreading diseases to humans [1,5,6]. Therefore, controlling rodent pest species is an important issue. The most common control techniques rely on lethal methods such as poison baiting and trapping [7]. Conventional control methods are associated with concerns about safety, specificity, animal welfare, and environmental impact. Consequently, there has been growing interest in developing more humane techniques that are cost-effective, species-specific, and environmentally friendly to control mouse populations [8].

In this regard, fertility control is an interesting alternative for controlling vertebrate pests, including mice [8,9,10,11]. Contraception by induced immunity (immunocontraception) is a fertility regulation method using the immune system to disrupt reproductive function and prevent fertilization [9,12,13]. Contraceptive vaccines evoke an immune response to molecules that have indispensable roles in the reproduction process. Several proteins have been used successfully to reduce the fertility of various animal species, including antigens associated with sperm [14,15,16] and oocytes [17,18,19], as well as hormones and their receptor proteins [20,21]. The unique specificity of sperm and oocyte proteins makes them ideal candidates for immunocontraceptive vaccines. The oocyte-specific protein zona pellucida (ZP), with a pivotal role in the process of fertilization, has been proposed as one of the most desirable targets for the development of contraceptive vaccines [13,22]. The ZP is a glycoprotein matrix that surrounds the plasma membrane of all mammalian oocytes. In mammals, the ZP is composed of either three or four glycoproteins. Antigens derived from the ZP protein subunits have successfully induced an immunocontraceptive effect in various species of mammals [12,23]. In mice, the ZP matrix consists of three glycoproteins conventionally known as ZP1, ZP2, and ZP3 [24,25,26]. ZP3 protein-based antigens have been reported to have a strong contraceptive effect and lead to reduced fertility in many species [27,28,29]. However, the lack of species specificity of protein-based vaccines limits their widespread use for wildlife population control [13,30]. Moreover, injectable immunocontraceptives are clearly not suitable for field applications for controlling the population of small- and large-scale dispersed animals such as wild mice. The availability of oral immunocontraceptive vaccines could increase the scope of contraceptive applications for wildlife population control and facilitate mouse population management.

Oral application of vaccine necessitates species specificity of contraceptive antigens to avoid affecting the fertility of non-target animals. The development of peptide-based contraceptive vaccines is an attractive approach for producing species-specific antigens [31,32,33]. Targeting rodent-specific peptides from areas of ZP proteins that are less conserved between species has been predicted to increase the target specificity of contraceptive vaccines [34,35,36]. In mice, the ZP3 protein plays a key role during fertilization as the primary receptor for sperm and the inducer of the sperm acrosome reaction [37,38,39]. A short glycopeptide of ZP3 corresponding to amino acid residues 328–342 (mZP3) has been suggested to be directly involved in these activities [38,40]. Vaccines based on the mZP3 contraceptive peptide appear to have the desired properties. First, immunization of mice with synthetic or recombinant forms of this peptide and other closely related peptides has reduced fertility in some mouse strains, including wild mice [15,41,42,43]. Second, it has not been detected immunologically in other species such as hamster, guinea pig, cat, or dog [41,44]. Therefore, the mZP3 peptide is considered a promising target for developing a mouse-specific contraceptive vaccine [32,43]. However, in many cases, peptide-based vaccines have lower immunogenicity than protein antigens [45]. Integrating adjuvants—such as a ‘promiscuous’ T-cell epitope from tetanus toxoid, which is reported to be a universal immunogenic epitope—is an approach towards improving the efficacy of peptide-based vaccines [46,47,48,49]. Furthermore, the use of repeated peptide epitopes has been shown to enhance the immunogenicity of contraceptive peptide-based vaccines [32,35,50].

Transgenic plants are a promising platform for producing oral vaccines. Plants offer several advantages compared to other available conventional systems, such as bacterial and mammalian cell-based production systems. Plant expression systems are considered rapid, cost-effective, and safe platforms for the large-scale production and delivery of vaccines [51,52]. In addition, plants have the ability to produce functional proteins with eukaryotic post-translational modifications including glycosylation, which are often required for the biological activity and function of many mammalian proteins [53,54]. Furthermore, plant expression systems provide safety benefits, as they greatly reduce the risk of contamination and transmission of mammalian pathogens from the host [55,56]. Nevertheless, the expression of small proteins can be a challenge to eukaryotic expression systems [57]. Several studies have illustrated the positive effect of protein fusion as a strategy to improve the production of recombinant proteins in plant production systems [58].

Plant-derived recombinant proteins can be expressed either by stable transformation or through transient expression. The latter approach provides for rapid production of high yields of recombinant proteins compared to time-consuming stable transformation [59]. MagnICON technology is a transient expression system based on a plant viral vector, with the potential of producing recombinant protein up to 80% of total soluble protein [60]. This system combines the advantages of different biological systems, including the efficient DNA delivery of *Agrobacterium tumefaciens*, the speed and high expression levels of a plant RNA virus, as well as posttranslational modifications and low cost of plant expression systems [61,62].

In this study, we explored the potential of plants for producing glycosylated putative contraceptive antigens using the MagnICON expression system in *N. benthamiana*. We chose a mouse-specific glycopeptide from ZP3 (mZP3, amino acids 328–342) as the contraceptive epitope for constructing the antigens. The T-cell help for eliciting an immune response was provided by inclusion of T-cell epitope from tetanus toxoid (TT). The objectives of this study were to evaluate the production of glycosylated mZP3-based antigens in *N. benthamiana* and to evaluate the capability of this small peptide to induce antibody responses by immunization with plant-produced antigens. Immunohistochemistry was performed using ovarian sections of wild mouse to assay antibody–oocyte interaction.

## 2. Materials and Methods

### 2.1. Construction of Plant Expression Vectors

**Figure 1:**
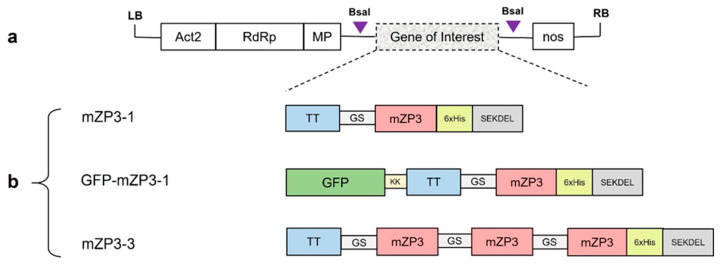
Schematic representations of the T-DNA of MagnICON and the expression constructs used for transient expression of the contraceptive mouse ZP3 peptide in the plant. (**a**) Binary ‘MagnICON’ vector used for transient expression. Act2, Arabidopsis actin 2 promoter; RdRp, RNA-dependent RNA polymerase; MP, TMV movement protein; nos, *A. tumefaciens* nopaline synthetase gene terminator. Triangles represent BsaI cleavage sites. (**b**) mZP3-1, GFP-mZP3-1, and mZP3-3 constructs used for transient expression. mZP3, mouse ZP3 peptide (aa residues 328–342) contains T-cell and B-cell epitopes; TT, T-cell epitope of tetanus toxoid (aa residues 830–844); GS, (GSSSS)3 flexible linker; 6xHis, 6 histidine residues; SEKDEL, ER retrieval signal; KK, dilysine linker.

The coding sequences of mZP3-1 (containing a copy of mZP3 peptide) and mZP3-3 (containing three copies of mZP3 peptide) constructs were designed and codon optimized for expression in *N. benthamiana* (Figure S1). The gene constructs were synthesized by Eurofins Genomics GmbH (Ebersberg, Germany) and cloned into the pEX-A vector. To construct GFP-mZP3-1, the green fluorescent protein (GFP) coding region was amplified from the vector pICH18711 [60] and fused to the 3′ end of the mZP3-1 by overlapping PCR. All coding regions were flanked by BsaI restriction sites using PCR amplification. The PCR products were cloned separately into the pJET1.2 cloning vector (CloneJET PCR Cloning Kit, Thermo Fisher Scientific, Waltham, MA, USA). Subsequently, the gene sequences were inserted into the TMV-based expression vector pICH31120 of the MagnICON system with BsaI restriction/ligation [63]. The MagnICON™ vectors were kindly provided by Nomad Bioscience (Halle/Saale, Germany). The binary MagnICON vector pICH31120 and the expression constructs are shown in Figure 1.

### 2.2. Agroinfiltration Procedure

Expression vectors with their cassettes were separately electroporated into *Agrobacterium tumefaciens* strain ICF320. Transformed colonies were inoculated into 5 mL Luria–Bertani (LB) media supplemented with 50 μg/mL kanamycin and 50 μg/mL rifampicin and incubated overnight at 28 °C in a shaking incubator (220 rpm). Two milliliters of the *Agrobacterium* suspensions were diluted into 200 mL cultures containing the same antibiotics and grown at 28 °C/220 rpm overnight. The *Agrobacterium* cells were harvested by centrifugation at 4560× *g* for 30 min at room temperature (RT) and resuspended in infiltration buffer (10 mM 2-(N-morpholino) ethanesulfonic acid (MES), pH 5.8, 10 mM MgSO_4_, 0.02% Silwet Gold) to a final OD_600_ of 0.15–0.2. Transient expression was carried out by vacuum-agroinfiltration of 6- to 8-week-old *N. benthamiana* grown in the greenhouse as described previously [64].

### 2.3. Total Soluble Protein Extraction from N. benthamiana Leaves

Agroinfiltrated *N. benthamiana* leaves were harvested at various days post-infiltration (dpi), lyophilized using a freeze dryer system and ground. Total soluble protein (TSP) was extracted from freeze-dried leaf material (25 mg) homogenized in 500 µL ice-cold extraction buffer using a Precellys 24 homogenizer (Bertin Instruments, Montigny-le-Bretonneux, France). The extract was clarified by repeated centrifugation at 15,000× *g* for 15 min at 4 °C. The supernatant was collected as the total soluble protein (TSP) fraction, and the protein concentration was measured using the Pierce™ Coomassie Plus (Bradford) assay kit (Thermo Fisher Scientific), with bovine serum albumin (BSA) as the standard.

### 2.4. Ni-NTA Purification

His-tagged recombinant mZP3-1, GFP-mZP3-1, and mZP3-3 were purified using immobilized metal affinity chromatography (IMAC) based on the affinity for the 6His purification tag. For large-scale protein extraction, 30 g of ground leaf material was homogenized in ice-cooled protein extraction buffer (50 mM NaH_2_PO_4_, 300 mM NaCl, 250 mM sucrose, 5 mM imidazole) using a Homogenizer (Polytron^®^ PT 2100, Kinematica AG, Malters, Switzerland) for 30 s at 30,000 rpm. Lysates were centrifuged at 4600× *g* for 15 min and then clarified by repeated ultracentrifugation at 16,000× *g* for 30 min at 4 °C. The supernatants were collected and loaded onto a column (Bio-Rad Laboratories, Hercules, CA, USA) containing pre-equilibrated Nuvia™ IMAC Ni-charged resin (Bio-Rad Laboratories, Hercules, CA, USA). The column was washed with washing buffer (50 mM NaH_2_PO_4_, 20 mM imidazole, 20 mM NaCl, pH 8). The target recombinant protein was further eluted with elution buffer (50 mM NaH_2_PO_4_, 300 mM imidazole, 300 mM NaCl, pH 8). The elution fraction was concentrated and desalted using Vivaspin 20 centrifugal concentrators with a 10 kDa cut-off membrane (Sartorius AG, Göttingen, Germany).

### 2.5. SDS–PAGE and Western Blot Analysis

Protein extracts were mixed with loading buffer (150 mM Tris, pH 6.8, 10% glycerin, 3% SDS, 1% β-mercaptoethanol, and 2.5% bromophenol blue) and denatured at 95 °C for 5 min. The proteins were separated under denaturing conditions using 12% SDS–PAGE and then electroblotted onto a nitrocellulose membrane (GE Healthcare Europe GmbH, Solingen, Germany) via wet transfer at 100 V for 30 min. Membranes were blocked for 2 h with 5% (*w/v*) nonfat milk powder in TBST, pH 7.6, containing 20 mM Tris, 150 mM NaCl, and 0.05% (*v/v*) Tween 20. After three washes with TBST, membranes were probed with mouse monoclonal anti-His antibodies (Dianova, Hamburg, Germany) (1:1000 dilution) for 2 h at RT. Following another washing step, the membranes were probed with horseradish peroxidase (HRP)-conjugated donkey anti-mouse antibodies (Dianova) (1:10,000 dilution) for 1 h at RT. After a final wash with TBS, proteins were detected with the enhanced chemiluminescence technique using a Kodak Biomax light X-ray film (VWR; Darmstadt, Germany). Concanavalin A (Con A) lectin blot analysis was conducted as previously described [64] to visualize the glycosylation of recombinant proteins.

### 2.6. Enzyme-Linked Immunosorbent Assay (ELISA)

The content of mZP3 peptide was determined using ELISA in 96-well microtiter plates. Briefly, the plate was coated with diluted leaf extract in carbonate buffer (pH 9.6) for 2 h at RT and washed three times with PBS containing 0.05% Tween 20 (PBST). The plate was blocked with 1X RotiBlock blocking solution (Carl Roth GmbH + Co. KG, Karlsruhe, Germany) for 1 h at RT and washed as previously described. Rabbit anti-mZP3 primary antibody (Bioserv, Rostock, Germany) was added at a 1:500 dilution in PBS, incubated at RT for 2 h, and then washed three times. The plate was then incubated for 1 h at RT with HRP-conjugated goat anti-rabbit secondary antibodies (Dianova) at a 1:2000 dilution in PBS. Tetramethylbenzidine (TMB) substrate was added to each well to develop the color in the dark for 10 min, and the reaction was stopped with 250 mM H_2_SO_4_. Absorbance was read at 450 nm.

### 2.7. Subcutaneous Immunization of Mice

The animal experiments were performed in accordance with the German animal protection regulations and were approved by the relevant authorities (Landesamt fur Landwirtschaft, Lebensmittelsicherheit und Fischerei, Mecklenburg-Vorpommern, Germany; permission No. 7221.3-1-073/17 and 7221.3-3.2-002/22).

Female BALB/c mice (6–8 weeks old) were divided into three groups with five mice in each group. Primary immunization was carried out on day 1 and two booster immunizations at three-week intervals. Injections were administered over the mouse neck. The first group received GFP-mZP3-1 (containing 50 µg mZP3), the second group received mZP3-3 (containing 50 µg mZP3), and the control group received PBS. All doses contained 10% Polygen (MVP Lab) as an adjuvant. Serum samples were collected before primary immunization and two to three weeks after each immunization. Female BALB/c mice were mated with proven fertile BALB/c males of similar age three weeks after the final immunization. Males were removed after 2 weeks, and the females were allowed to litter.

### 2.8. Detection of the Antibody Titer Using ELISA

The specific IgG responses in serum were determined using endpoint titer ELISA. Briefly, a 96-well plate was coated with 50 μL of plant-produced mZP3 (0.5 µg per well) and incubated at RT for 2 h. Nonspecific binding was prevented by incubation in PBS buffer containing 1% (*w/v*) BSA for 1 h at RT. The plates were washed three times with PBST and incubated with serum samples (50 µL per well) serially diluted in PBS at RT for 2 h. Then, the plate was incubated with HRP-conjugated donkey anti-mouse secondary antibodies (Dianova, Hamburg, Germany) at a dilution of 1:2000 in PBS at 37 °C for 1 h. The plate was washed, and the color was developed with TMB substrate solution (100 μL per well). After 12 min, the reaction was terminated by adding 100 µL of 250 mM H_2_SO_4_. Absorbance values were determined at 450 nm.

### 2.9. Indirect Immunofluorescence

Mouse ovarian sections were provided by BIOSERV Analytik (Rostock, Germany). Sections were deparaffinized and subjected to antigen retrieval using sodium citrate buffer (10 mM sodium citrate, 0.05% Tween 20, pH 6.0). Sections were blocked with 10% goat serum blocking solution (Life Technologies, Frederick, MD, USA) at RT for 1 h, rinsed with PBS, and incubated in 1× mouse-on-mouse IgG blocking solution (Invitrogen, Thermo Fisher Scientific, Waltham, MA, USA) at RT for 1 h. Sections were incubated with a 1:30 dilution of serum samples and kept at 4 °C overnight. The sections were washed and incubated with 10 µg/mL fluorescein isothiocyanate (FITC)-conjugated goat anti-mouse IgG (Invitrogen) as a secondary antibody for 1.5 h at 37 °C. After washing, the slides were mounted with DAPCO mounting medium (25 mg/mL DABCO, 90% glycerol, 10% PBS, pH 8.5) and observed under a fluorescence microscope. The sections were stained with methylene blue for bright-field microscopy.

### 2.10. Histological Analysis

On day 99 of the experiment, one mouse from each group was sacrificed. The ovaries were collected, fixed in 4% paraformaldehyde and then paraffin embedded. The ovaries were sectioned at 5 µm, stained with hematoxylin and eosin, and observed under a microscope.

### 2.11. Statistical Analysis

The experimental data were analyzed using IBM SPSS statistical software version 27. The comparison of means was performed using one-way analysis of variance (ANOVA). Duncan’s test was used as post-hoc test to measure specific differences between the means. The statistically significant differences are shown with one asterisk (*), indicating *p* values < 0.05, and two asterisks (**), indicating *p* values < 0.001.

## 3. Results

### 3.1. Construction of Expression Vectors for Transient Expression

The mouse zona pellucida 3 peptide CSNSSSSNSSSSQFQ (mZP3), corresponding to amino acids 328–342, contains a T-cell and a B-cell epitope [41,42,43]. To assess the expression of mZP3 in plants and immunogenicity of plant-made recombinant mZP3 in mice, three gene constructs (mZP3-1, GFP-mZP3-1, and mZP3-3) were developed (Figure 1b). In the mZP3-1 construct, a promiscuous T-cell epitope (aa residues 830–844) of TT was fused at the N-terminus of the mZP3 sequence using a flexible GS linker to enhance the immunogenicity of the antigen [48]. To facilitate the purification of recombinant protein, a 6×His tag was fused at the C-terminal end of mZP3. The retention in the endoplasmic reticulum (ER) was achieved through the addition of a SEKDEL retention signal at the C-terminus of the construct. The GFP-mZP3-1 construct was developed by fusing the mZP3-1 construct to GFP via a dilysine linker (KK). In the mZP3-3 construct, the antigenic peptide was tripled by linking three repeated copies of mZP3 via GS linkers. All three resulting constructs were codon optimized for *N. benthamiana* to increase protein expression and cloned into the MagnICON-based plant expression vector pICH31120 to target the expression of proteins into the ER.

### 3.2. Optimization of mZP3 Expression in N. benthamiana

**Figure 2:**
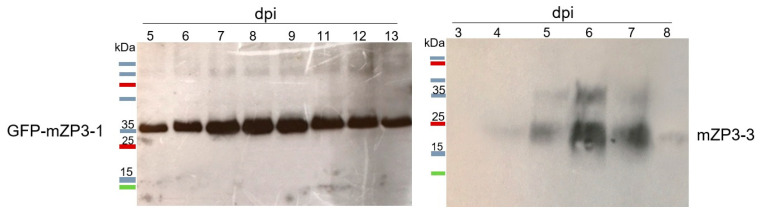
Western blot of *N. benthamiana* crude leaf extracts to detect protein expression and to determine the optimal harvest time. Expression of GFP-mZP3-1 (**left**) and mZP3-3 (**right**) in the infiltrated leaves, detected by western blot of 30 µg TSP, probed with anti-His monoclonal antibodies. dpi, days post-infiltration. Blots cropped for clarity, for uncropped blots see Figure S3.

*N. benthamiana* plants were infiltrated with the resulting vectors in three independent repetitions. Protein expression was examined following transient expression in *Agrobacterium*-infiltrated leaves. The leaves were harvested at different days post-infiltration (dpi), and total soluble proteins were extracted and determined with a Bradford assay. Time-course evaluation of protein expression levels of each construct was performed using western blot probed with anti-His monoclonal antibodies. The optimal harvest time of recombinant proteins was determined based on the accumulation of target proteins and morphology of the infiltrated leaves. Western blot analysis of TSP from leaves infiltrated with mZP3-1 showed no protein band corresponding to the expected size of ~8 kDa for the mZP3-1 protein (Figure S2). Accordingly, no band at the expected size for the non-glycosylated forms of GFP-mZP3-1 and mZP3-3 (34.7 and 11 kDa, respectively) could be detected (Figure 2). However, a strong band representing a protein with a molecular mass of ~37 kDa was detected in the total soluble protein extracts from leaves infiltrated with GFP-mZP3-1. Protein accumulation was observed in all selected leaves, reached the highest level at 8 dpi, and gradually declined thereafter (Figure 2, left). In western blot analysis of crude extract from mZP3-3-expressing leaves, a ∼18 kDa protein band was detected. Accumulation of the protein was observed from 5 dpi to 7 dpi, while the protein was barely detectable before and after this time (Figure 2, right). A higher molecular mass band (~36 kDa) was also presented, which appeared to be the dimer form of the protein.

**Figure 3:**
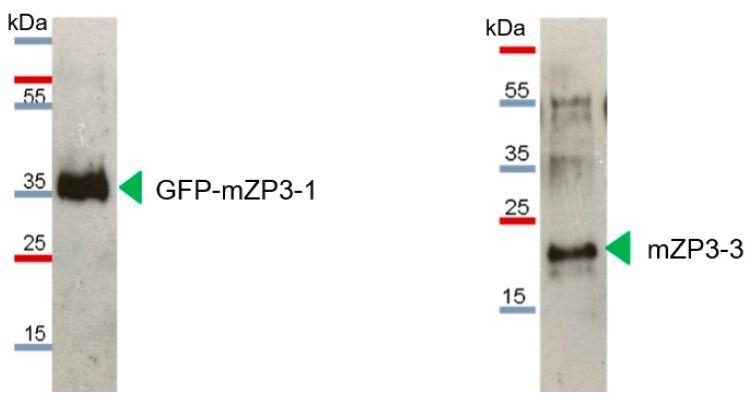
Profile of lectin binding to the *N. benthamiana*-expressed proteins to detect the presence of sugar residues on proteins. The profile represents the binding of Con A to the recombinant GFP-mZP3-1 (**left**) and mZP3-3 (**right**). Lectin blot labeled with peroxidase-conjugated concanavalin A. Blots cropped for clarity, for uncropped blots see Figure S4.

The plant-produced proteins showed higher molecular masses than those expected for unglycosylated proteins. This finding pointed to the possible addition of glycan chains during expression in plants and retention in the ER, since the mZP3 peptide carries two potential glycosylation sites. The presence of glycans on the plant-produced GFP-mZP3-1 and mZP3-3 proteins was assessed with lectin blotting using peroxidase-conjugated concanavalin A (Con A), which has a high affinity for binding to sugar residues. Lectin blots of recombinant GFP-mZP3-1 and mZP3-3 probed with Con A identified carbohydrates and revealed dominant bands of ~37 kDa and ~18 kDa, respectively (Figure 3). This result indicated that the observed protein bands corresponded to glycosylated forms of the GFP-mZP3-1 and mZP3-3 recombinant proteins.

Protein analysis showed that GFP-mZP3-1 and mZP3-3 targeted to the ER were successfully produced in *N. benthamiana* plants. Based on the plant morphology and highest accumulation of proteins in plants, the optimal harvest time for GFP-mZP3-1- and mZP3-3-infiltrated leaves was determined to be approximately 8 dpi and 6 dpi, respectively.

**Figure 4:**
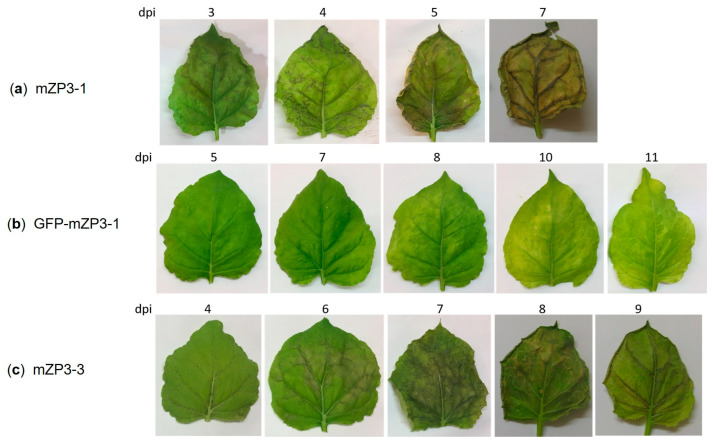
Morphological changes in *N. benthamiana* leaves agroinfiltrated with pICH31120 vector carrying mZP3-1 (**a**), GFP-mZP3-1 (**b**), or mZP3-3 (**c**) constructs. Protein expression induced chlorosis/necrosis and progressed to cell death on different days. Numbers represent days post-infiltration (dpi).

All infiltrated plants displayed morphological disorders from chlorosis to necrosis and finally leaf death, although at different dpi. In the case of mZP3-1, necrosis started on day 3 and increased rapidly, resulting in plant death by day 7 (Figure 4a). However, when the plants were infiltrated with the GFP-mZP3-1 fusion construct, the phenotypic effects were less pronounced, and symptoms of chlorosis began on day 7 (Figure 4b). In plants infiltrated with mZP3-3, leaves started to show necrosis at 4 dpi. Wilting was observed on day 7 and resulted in plant death by day 10 (Figure 4c). Expression of mZP3-3 caused moderate leaf necrosis compared to mZP3-1, although it was more severe than that of GFP-mZP3-1 expression.

### 3.3. Purification and Characterization of Plant-Produced mZP3 Antigens

**Figure 5:**
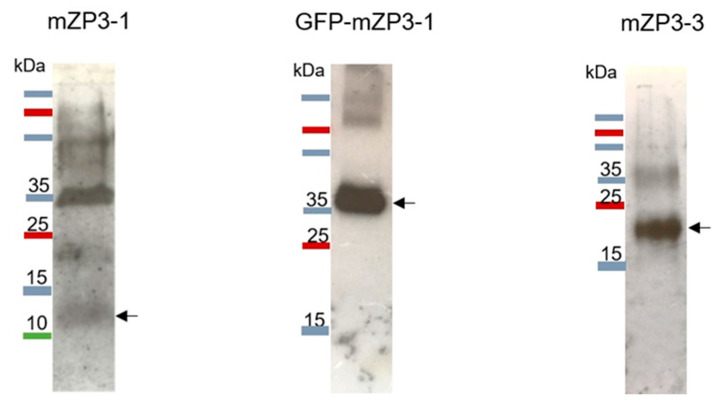
Detection of purified plant-expressed recombinant proteins using Ni-NTA affinity chromatography. Western blot of the 30× concentrated elution fraction from mZP3-1 in comparison to the unconcentrated elution fraction from GFP-mZP3-1 and mZP3-3. Arrows indicate the bands at the expected size of the glycosylated proteins. Western blots probed with mouse anti-His monoclonal antibodies. Blots cropped for clarity, for uncropped blots see Figure S5. For Coomassie-stained SDS-PAGE of purified plant-expressed recombinant proteins, see Figure S6.

Purification of plant-produced mZP3-1, GFP-mZP3-1, and mZP3-3 proteins was carried out under non-denaturing conditions through Ni^2+^ -charged column chromatography. Fractions were collected and subjected to SDS–PAGE followed by western blots probed with mouse anti-His monoclonal antibodies. Although mZP3-1 was not detectable in crude protein extracts, analysis of the concentrated purified protein from the chromatography column revealed bands of proteins that confirmed recombinant expression of mZP3-1 (Figure 5). The 12 kDa band detected by western blot corresponds to the expected size of the glycosylated mZP3-1 protein; however, possible glycosylation could not be detected by lectin blotting. Bands with higher molecular mass were also observed in the western blot, which are close to the molecular mass of oligomer forms of the mZP3-1 protein. The infiltrated plant leaves showed the highest expression of mZP3-1 at day 4 post infiltration; however, the yield of mZP3-1 was low compared to that of the other two proteins. Figure 5 illustrates 30× concentrated purified mZP3-1 compared to 1× purified GFP-mZP3-1 and mZP3-3 proteins.

The concentration of plant-made mZP3 peptide in crude protein extract and in antigens purified from *N. benthamiana* infiltrated leaves was quantified with ELISA. Compared with mZP3-1, GFP-mZP3-1 and mZP3-3 increased the production of the mZP3 peptide by more than 20 and 25 times, respectively (Table 1). However, among the three purified proteins, the highest concentration of mZP3 peptide was obtained in GFP-mZP3-1 (53.4 ± 1 µg/g DW). The purified mZP3-1 and mZP3-3 revealed 3.4 ± 0.8 µg/g DW and 32.7 ± 2.3 µg/g DW mZP3 peptide, respectively (Table 1). The accumulation of mZP3-1 was not sufficient for application in mouse vaccination. Therefore, only GFP-mZP3-1 and mZP3-3 were purified on a large scale from *N. benthamiana* leaves at the optimal harvest time and prepared for vaccination of female BALB/c mice. Ni-NTA affinity purified proteins were concentrated and desalted using Vivaspin™ ultrafiltration spin columns. Impurities were removed from samples using a 0.22 µm syringe filter.

### 3.4. Immunogenicity of mZP3-Based Antigens

**Figure 6:**
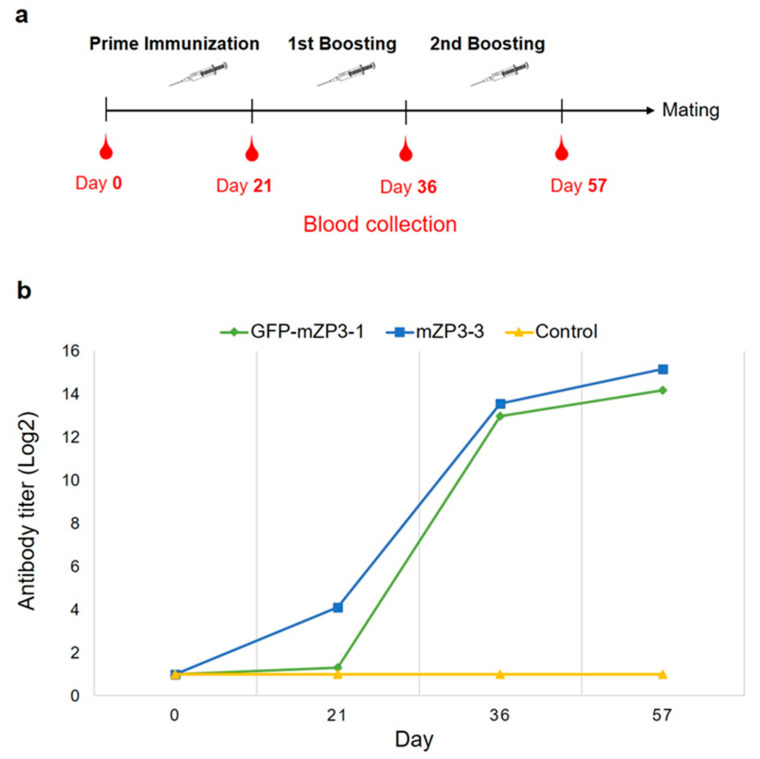
Kinetics of serum antibody responses in female BALB/c mice following prime-boost parenteral immunization. The mice were separated into three groups; two experimental groups were immunized with 50 µg of ZP3 peptide, in the form of GFP-mZP3-1 or mZP3-3, along with Polygen adjuvant, and the Control group was immunized with PBS adjuvanted by Polygen. (**a**) Schematic representation of the immunization protocol and blood collection. (**b**) The endpoint titers of anti-mZP3 antibodies in BALB/c mice immunized with plant-produced GFP-mZP3-1 (♦), mZP3-3 (■), or PBS as a control (▲) on days 0, 21, 36, and 57. The values are the means log2 endpoint titers of all mice in each group (*N* = 5).

To determine whether the plant-produced mZP3-based antigens are immunogenic in mice, a prime-boost immunization assay was conducted (Figure 6a). Two groups of five female BALB/c mice were immunized and boosted two times at three-week intervals with either recombinant GFP-mZP3-1 or mZP3-3 containing 50 µg mZP3 peptide. Additionally, the third group of five mice received PBS as a control. The antibody responses of immunized mice were assessed using ELISA. Figure 6b illustrates the mean endpoint titer of IgG antibodies in pre-immune sera and sera of the immunized mice after each immunization. Although an immune response was not observed after prime injection in GFP-mZP3-1-immunized mice, serum antibody responses increased greatly after the first booster and showed a significant difference compared to the control group. All GFP-mZP3-1-immunized mice produced detectable IgG antibodies at serum dilutions up to 1:8000 (log2, 12.96) after the first boost dose. Antibody levels continued to increase in all vaccinated mice following the second boost dose. However, there were no detectable IgG antibody titers in all sera of control mice throughout the whole course (Figure 6b).

In mice immunized with plant-produced mZP3-3, only one mouse produced detectable IgG antibodies at a 1:4000 dilution after the priming dose. However, antibody titers increased drastically in the remaining animals after the first boost dose. Although there was variation between the IgG titers of individuals, antibody responses were detectable in all mZP3-3-immunized animals at serum dilutions higher than 1:8000. The antibody titer subsequently increased in all mice following the second booster (Figure 6b). Two mice showed antibody responses even up to a titer of 1:64,000 (log2, 15.96) after the second boost injection.

**Figure 7:**
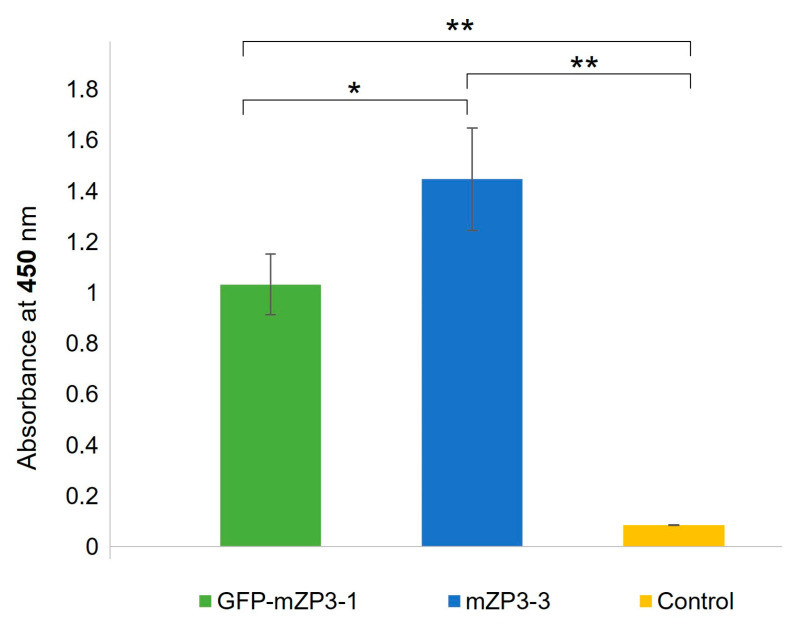
Comparison of serum antibody responses in mice immunized with GFP-mZP3-1, mZP3-3, or PBS after the final boost injection. Absorbance values are shown for sera of each immunized group at a 1:1000 dilution. Data are presented as the mean ± SD absorbance values at 450 nm. * *p* < 0.05; ** *p* < 0.001.

These results indicated that both plant-produced antigens were capable of inducing antibodies against mZP3, which increased following each injection and reached the peak after the final boost dose. Figure 7 represents the comparison of antibody responses in mice from the three groups after the final boost injection at a 1:1000 dilution. The results showed a significant difference not only between the experimental groups and control (*p* < 0.001) but also between the experimental groups (Figure 7). Mice immunized with the plant-produced mZP3-3 antigen elicited a significantly higher antibody response (*p* < 0.05) than those immunized with the GFP-mZP3-1 antigen after the second booster.

### 3.5. Fertility and Ovarian Effects in BALB/c Mice Immunized with mZP3 Antigens

To assess the fertility of female mice immunized with GFP-ZP3-1 or mZP3-3 antigens or PBS, individuals were mated with BALB/c males of similar age three weeks after the final immunization. Female mice were allowed to produce litters. The results showed that there was no significant reduction in the fertility or litter size of mice immunized with plant-produced GFP-ZP3-1 or mZP3-3 antigens. All animals in the treated groups and control group were fertile. No significant reduction in the mean litter size of mice immunized with recombinant GFP-mZP3-1 was achieved (Table 2). Although one animal from the mZP3-3 group showed a reduction in litter size, the mean litter size of this group was not significantly different from that of the control group (Table 2). The mean litter sizes of mice immunized with GFP-mZP3-1 (5.7 ± 0.6) and mZP3-3 (7 ± 1.5) were not significantly different from those of control mice (7 ± 0.9).

**Figure 8:**
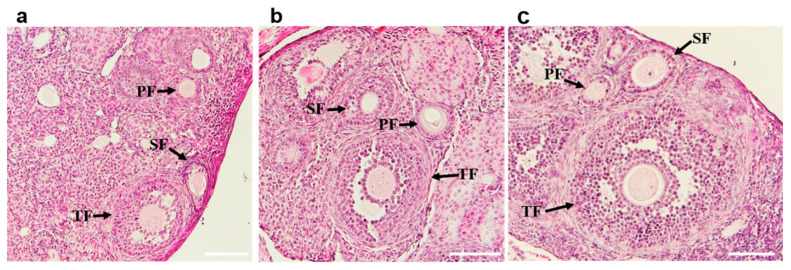
Histological analysis of the hematoxylin and eosin-stained ovarian sections of BALB/c mice. Ovaries of the mice immunized with GFP-mZP3-1 (**a**) and mZP3-3 (**b**) showing normal follicles at different stages of development as in ovaries from mouse immunized with PBS (**c**). PF, primary follicles; SF, secondary follicles; TF, tertiary (antral) follicles. Scale bars = 100 µm.

Histological examination of the ovaries of immunized mice showed no evidence of oophoritis or disrupted folliculogenesis. The ovaries demonstrated normal follicles at different stages of development (Figure 8).

### 3.6. Reactivity of Serum Antibodies with the Mouse ZP Matrix by Indirect Immunofluorescence

**Figure 9:**
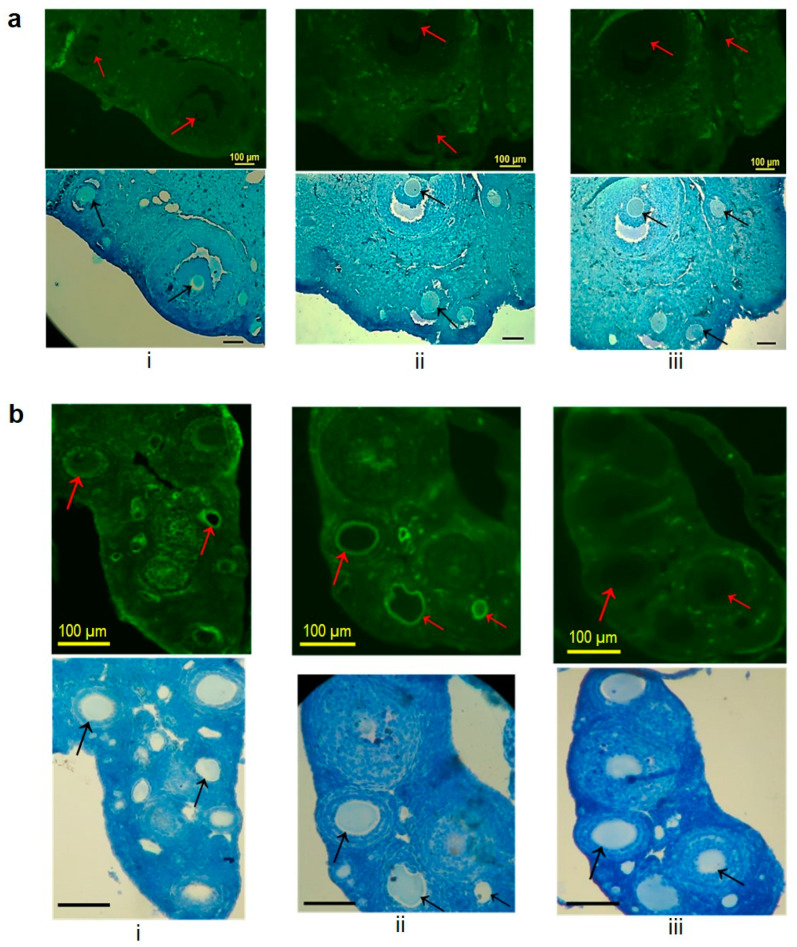
Indirect immunofluorescence microscopy for detecting the reactivity of serum antibodies with zona pellucida matrix in ovarian sections. Ovarian sections from BALB/c mouse (**a**) and wild mouse (**b**) treated with sera from mice immunized with GFP-mZP3-1 (i) and mZP3-3 (ii). Control experiments were performed with pre-immune sera of mZP3-3-immunized mice (iii). The upper pictures show sections in the fluorescent field, and the lower pictures represent stained sections in the bright field. Arrows represent the zona pellucida. Scale bars = 100 µm.

An indirect immunofluorescence assay was conducted to evaluate the binding of serum antibodies to the ZP matrix in BALB/c and wild mice. The results showed that the antibodies generated against the plant-produced GFP-mZP3-1 or mZP3-3 did not react with the ZP matrix surrounding the oocytes in BALB/c mice. Fluorescence signals from immune sera were similar to those of pre-immune serum samples (Figure 9a). However, immunofluorescence studies with wild mouse ovarian sections showed that serum antibodies from both immunized groups recognized the ZP matrix. Immune sera of immunized mice bound to the ZP of wild mouse ovarian sections, whereas pre-immune serum samples from the same animal did not show reactivity to the ZP matrix (Figure 9b).

## 4. Discussion

In the face of an overpopulation of mice, developing a species-specific, inexpensive, effective, and readily applicable contraceptive vaccine is required. This study aimed to develop an immunocontraceptive vaccine based on the mZP3 glycopeptide in plants.

To develop contraceptive vaccines targeting wild mouse populations, an effective mouse-specific antigen is needed. Vaccines based on the mZP3 peptide appear to be promising since the peptide has been found to be sufficiently immunogenic and contraceptive in wild mice [43]. In addition, it is hypothesized to be mouse-specific because it has not been detected in other species such as hamster, guinea pig, cat, or dog [41,44]. Therefore, it might be a good choice for producing a mouse-specific contraceptive vaccine in transgenic plants that is a suitable platform for the rapid, low-cost, and large-scale production of vaccines [56,65]. Moreover, transgenic plants can facilitate the administration of vaccines by providing a potential platform for the oral delivery of vaccines (52), which could be an ideal method for vaccination of wildlife populations.

To create an effective vaccine, it is necessary to produce a sufficient amount of stable antigen. Our results indicated that mZP3-1 was unstable, with accumulation levels dropping four days after infiltration, before the optimal day proposed for the expression system [60,66]. The low mZP3-1 yield might be related to its susceptibility to proteolytic processes in the heterologous environment [58,67,68]. Fusing recombinant peptides or proteins with a partner that is stably expressed in plants is one approach to increase the yield of target proteins in plants [69]. Fusion of mZP3-1 to the GFP coding region, resulting in GFP-mZP3-1, led to a more than 25-fold higher accumulation of mZP3. GFP has already been used successfully to improve protein production and stability under various conditions [70,71,72]. This stabilization seems to depend not only on the GFP sequence, but also on the protein size since mZP3-3 showed a similar effect on the stability and accumulation of the protein.

The low amounts of mZP3-1 could also be due to the massive damage that mZP3-1 production caused in the plants. It has already been observed that transient expression of ER-targeted proteins can put stress on plants and cause prohibitive levels of tissue necrosis that consequently lead to low yields of recombinant proteins [73,74,75]. Expression of GFP-mZP3-1 and mZP3-3 reduced the severity of tissue necrosis and resulted in higher protein accumulation. Other studies have achieved an increase in target protein accumulation by reducing the ER stress response and moderating plant tissue necrosis [73,76]. Hence, differences in protein accumulation might be due not only to differences in protein stability, but also to differences in the induction of plant stress responses.

The other necessity for an efficient vaccine is sufficient immunogenicity. Immunization of mice with mZP3-3 antigens led to the generation of significantly higher antibody responses against mZP3 compared to GFP-mZP3-1. In line with this, previous studies have shown that antigens containing multiple repeats of ZP3 epitopes stimulate higher antibodies in BALB/c mice compared with those containing single mZP3 epitopes [32,77]. Incorporation of multiple copies of epitopes into antigens has been investigated previously for various weak immunogens to enhance their immunogenicity [78,79,80].

Notably, the production of mZP3 antigens in plants seems to increase immunogenicity compared to other production systems. *E. coli*-produced single mZP3-peptide antigens elicited no significant antibody responses to ZP3 in BALB/c immunized mice [32], and ZP3 protein expressed in the insect cell-expression system [81] seems to induce lower levels of antibodies against mZP3 peptide compared to our study. These observations highlight the importance of the expression system for the production of recombinant ZP3 antigens and show the superiority of plants over other expression platforms in this case. The importance of glycosylation in the efficiency of recombinant mouse ZP3 antigens as immunocontraceptive has been already demonstrated [81]. Plants offer the ability to perform posttranslational modification of proteins in a similar, but not equal, way compared to mammalian proteins that can support the immunogenicity of plant-made vaccines [51,67,82]. Although glycosylation analysis was not carried out, the strong reaction of plant-produced proteins with the carbohydrate-binding protein Con A and the size of the proteins may be evidence for the glycosylation of the mZP3 peptide. Therefore, oligosaccharide residues present in plant-produced mZP3 may be a contributing factor in increasing the immunogenicity of antigens in vaccinated mice. Nevertheless, it might also indicate the stimulating effects of the TT epitope as an adjuvant. It has been observed previously that the antibody responses to ZP3 improved when additional appropriate T-cell helper epitopes were included in antigens [10,83].

Wild mice cause huge problems in immunization and mating studies due to their aggressive behavior. Hence, we had to restrict studies with live animals to the laboratory BALB/c mice used in most previous studies. Accordingly, despite high anti-mZP3 antibody titers, reproduction rates were not significantly reduced in immunized mice. It has been suggested that contraception depends on additional factors, such as mouse genetic background in combination with immune responses [32,43]. However, mZP3 peptide has been effective in reducing fertility in wild mice [43]. Moreover, the infertility of mice correlated with the reaction of antibodies to the zona pellucida. Hence, the immunological cross-reactivity between serum antibodies and wild mouse ZP observed in our study may reflect the ability of anti-mZP3 antibodies to bind to the ZP *in vivo* in wild mice. Therefore, further research is required to evaluate the contraceptive effect of plant-made mZP3 in wild mice.

In conclusion, the present study demonstrates that mZP3 can be effectively produced in *N. benthamiana* plants using a transient expression system. Compared to other production systems, plants seem to be more promising for producing highly immunogenic mZP3 antigens. Among the antigens studied, mZP3-3 is the best choice because it was efficiently expressed in the plant and induced high levels of antibodies that can react to the native zona pellucida of wild mice as the target species. The study is limited by the lack of information on the effect of plant-produced mZP3 in reducing the fertility of wild mice. Therefore, further research will be conducted to determine the contraceptive efficiency of plant-made mZP3-based antigens in wild mice. Furthermore, the practicality of injectable vaccines for wild pest animals is limited, so there is a need to formulate the plant-made mZP3 as effective bait for oral vaccination of mice.

## Figures and Tables

**Table 1:** mZP3 accumulation levels in crude plant extract and in purified antigens, as determined using ELISA.

Antigen	Time Point	mZP3 Peptide/TSP (%)	Purified mZP3 (µg/g LDW)
mZP3-1	4 dpi	0.047 ± 0.006	3.4 ± 0.8
GFP-mZP3-1	8 dpi	0.95 ± 0.05	53.4 ± 1
mZP3-3	6 dpi	1.18 ± 0.12	32.7 ± 2.3

Infiltrated *N. benthamiana* leaves were collected at the optimal time point and evaluated for mZP3 expression by ELISA. Data are reported as the mean ± SEM from three independent infiltrated samples. dpi, days post-infiltration; TSP, total soluble protein; LDW, leaf dry weight.

**Table 2:** Fertility of BALB/c mice immunized with recombinant mZP3-based antigens.

Antigen	Total Mice	Number of Injection	Number of Fertile Mice	Litter Size (Mean ± SEM)	*p*
GFP-mZP3-1	5	3	5/5	5.7 ± 0.6	N/S
mZP3-3	5	3	5/5	7 ± 1.5	N/S
PBS	5	3	5/5	7 ± 0.9	

Female BALB/c mice were immunized and boosted two times at three-week intervals with antigens in Polygen adjuvant and mated three weeks after the final boost. Values are means ± SEM. The *p* value indicates a significant difference (*p* < 0.05) between the treated and control groups. N/S, not significant.

## Data Availability

The datasets generated during this study are available from the corresponding author upon request.

## Supplementary Materials

The following supporting information can be downloaded at: https://www.mdpi.com/article/10.3390/vaccines11010153/s1. The following information is available. Figure S1: Protein and nucleotide sequences codon-optimized for *N. benthamiana*. Figure S2: Western blot of crude leaf extracts of *N. benthamiana* leaves infiltrated with mZP3-1. Figure S3: Western analysis of plant-produced GFP-mZP3-1 and mZP3-3. Figure S4: Profile of lectin binding to the *N. benthamiana*-expressed proteins. Figure S5: Detection of purified plant-expressed recombinant proteins. Figure S6: Coomassie-stained SDS-PAGE of purified plant-expressed recombinant proteins.
