# Supplementary material for: Plant-Produced Mouse-Specific Zona Pellucida 3 Peptide Induces Immune Responses in Mice
Source: Vaccines (Basel). 2023 Jan 10;11(1):153. doi: 10.3390/vaccines11010153 (PMC9866649; doi:10.3390/vaccines11010153)
Supplement: Supplementary file 1 [file vaccines-11-00153-s001.zip › vaccines-2118446-supplementary.pdf]

<u>**Supplementary Material**</u>

Plant-produced mouse-specific zona pellucida 3 peptide induces immune responses in mice

a

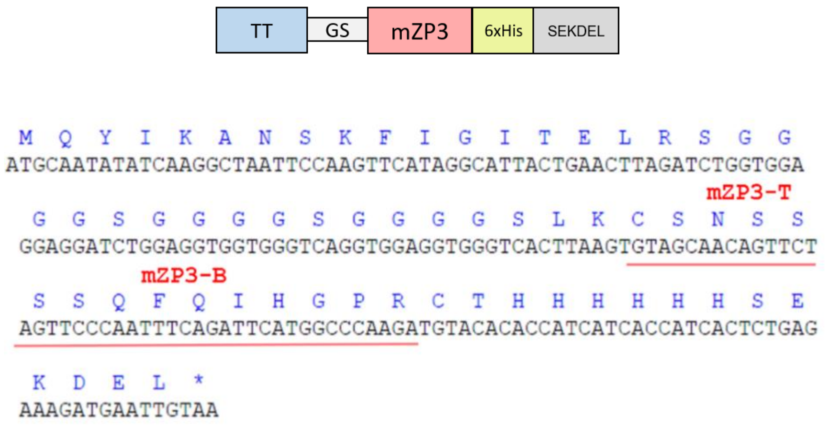

b

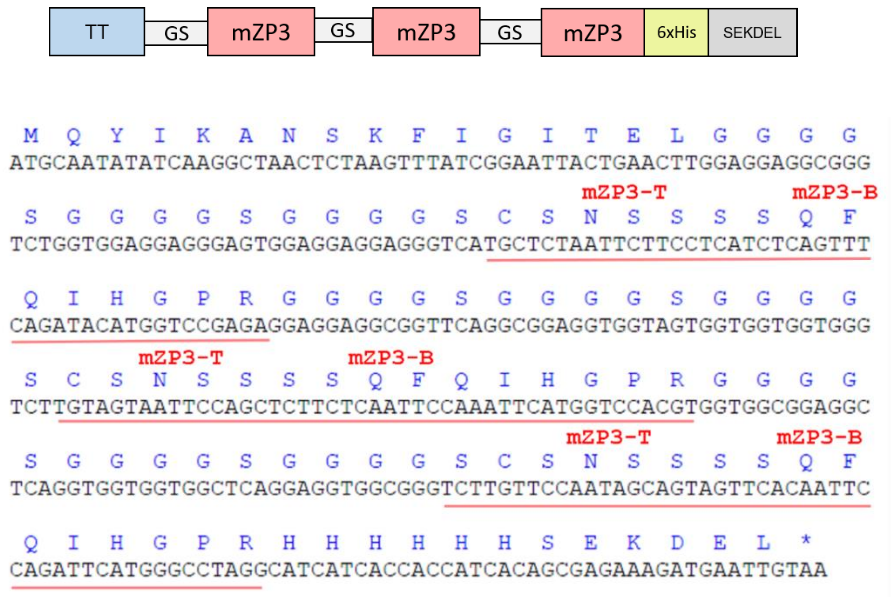

Figure S1. Protein and nucleotide sequences codon-optimized for *N. benthamiana*. The nucleotide and amino acid sequences of synthetized mZP3-1 (**a**) and mZP3-3 constructs (**b**). Nucleotides are represented in black and amino acids in blue. Mouse Zona pellucida 3-peptide (mZP3) sequences are underlined. The locations of the mouse ZP3 contraceptive T and B cell epitopes (mZP3-T and mZP3-B) are shown above the sequences.

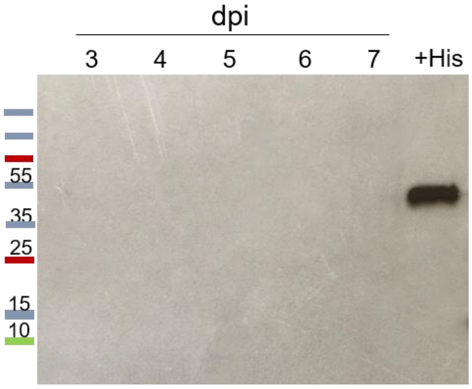

Figure S2. Western blot of crude leaf extracts of *N. benthamiana* leaves infiltrated with mZP3-1. No protein band corresponding to the expected size of the mZP3-1 protein was detected. Western blot was probed with anti-His monoclonal antibody. +His, a His-tagged protein as a positive western blot control. dpi, days post infiltration.

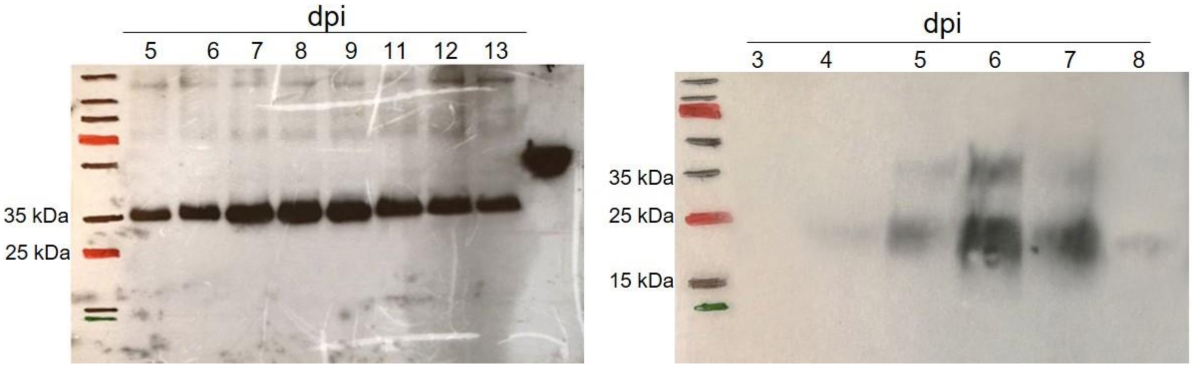

Figure S3. Western analysis of plant-produced GFP-mZP3-1 (left) and mZP3-3 (right). Figure 2 was cropped from these western blot images. dpi, days post infiltration.

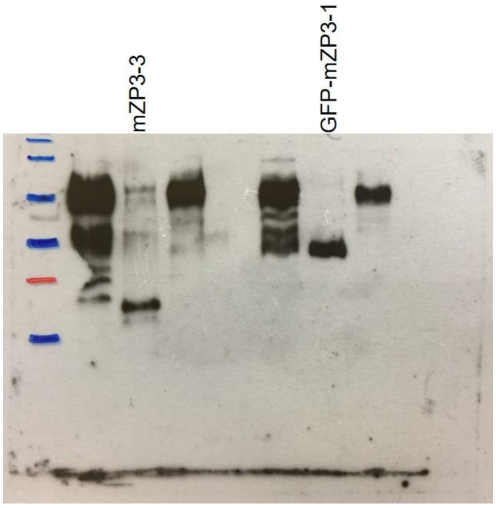

Figure S4. Profile of lectin binding to the *N. benthamiana*-expressed proteins. The panels of figure 3 were cropped from this lectin blot image.

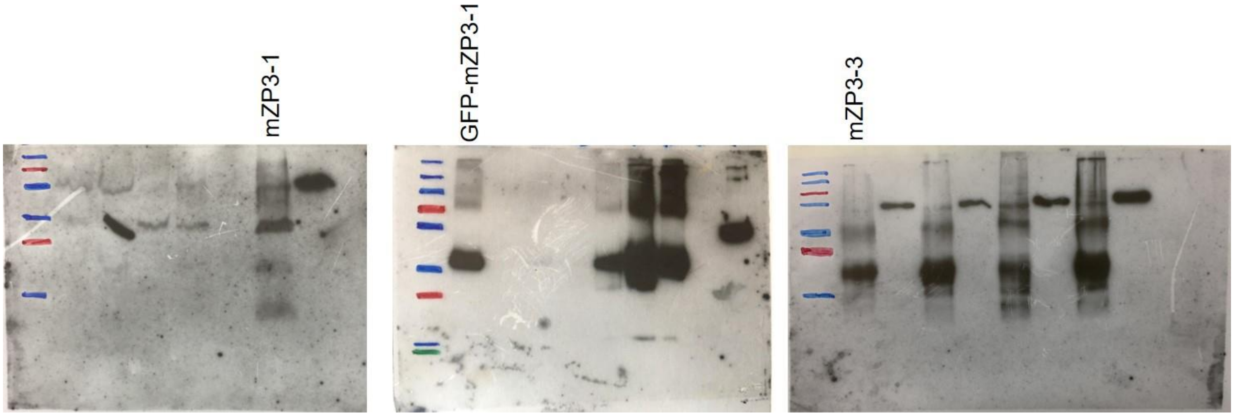

Figure S5. Detection of purified plant-expressed recombinant proteins. The panels of figure 5 were cropped from these western blot images.

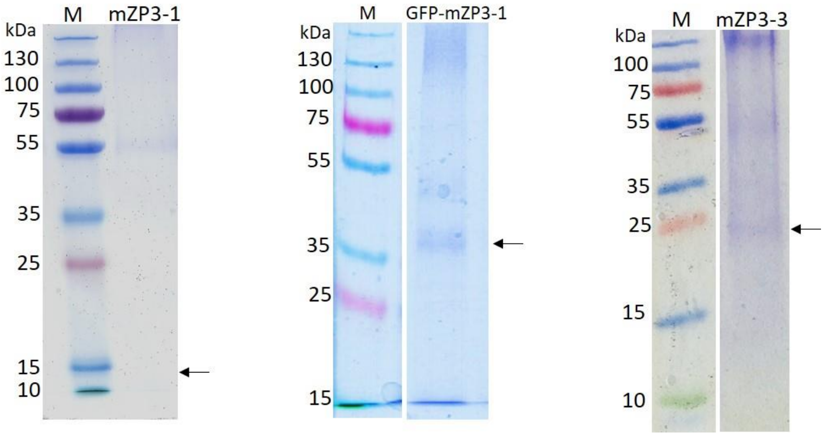

Figure S6. Coomassie-stained SDS-PAGE of purified plant-expressed recombinant proteins. M, Marker. Arrows indicate the expected size of the proteins.
